# Dealcoholization of Unfiltered and Filtered Lager Beer by Hollow Fiber Polyelectrolyte Multilayer Nanofiltration Membranes—The Effect of Ion Rejection

**DOI:** 10.3390/membranes13030283

**Published:** 2023-02-27

**Authors:** Áron Bóna, Áron Varga, Ildikó Galambos, Nándor Nemestóthy

**Affiliations:** 1 Soós Ernő Research and Development Center, University of Pannonia, Vár u. 8, H-8800 Nagykanizsa, Hungary; 2 Department of Research and Development, Pécsi Brewery, Alkotmány utca 94, H-7624 Pécs, Hungary; 3 Research Institute on Bioengineering, Membrane Technology and Energetics, University of Pannonia, Egyetem u. 10, H-8200 Veszprém, Hungary

**Keywords:** beer dealcoholization, nanofiltration, polyelectrolyte multilayer membrane, layer-by-layer membrane, membrane-based food processing, membrane modification

## Abstract

Membrane-based beverage dealcoholization is a successful process for producing low- and non-alcoholic beer and represents a fast-growing industry. Polyamide NF and RO membranes are commonly applied for this process. Polyelectrolyte multilayer (PEM) NF membranes are emerging as industrially relevant species, and their unique properties (usually hollow fiber geometry, high and tunable selectivity, low fouling) underlines the importance of testing them in the food industry as well. To test PEM NF membranes for beer dealcoholization at a small pilot scale, we dealcoholized filtered and unfiltered lager beer with the tightest available commercial polyelectrolyte multilayer NF membrane (NX Filtration dNF40), which has a MWCO = 400 Da, which is quite high for these purposes. Dealcoholization is possible with a reasonable flux (10 L/m^2^h) at low pressures (5–8.6 bar) with a real extract loss of 15–18% and an alcohol passage of ~100%. Inorganic salt passage is high (which is typical for PEM NF membranes), which greatly affected beer flavor. During the dealcoholization process, the membrane underwent changes which substantially increased its salt rejection values (MgSO_4_ passage decreased fourfold) while permeance loss was minimal (less than 10%). According to our sensory evaluation, the process yielded an acceptable tasting beer which could be greatly enhanced by the addition of the lost salts and glycerol.

## 1. Introduction

### 1.1. Low-Alcohol Beer

The non-alcoholic and low-alcohol beer market has steadily grown in recent years, driven by factors, such as health, legal restrictions, and social regulations. Consumers seeking non-alcoholic beer alternatives, mainly for health reasons, look for those with sensory characteristics, such as flavor, that are similar to conventional beer [1,2,3,4,5]. The alcohol-free beer market is reliably outperforming the one of total beer, with an annual global market growth of 7.5% [6].

Modified fermentation processes employed in the production of low-alcohol beer are limited fermentation and the utilization of specialized yeast strains. These processes are commonly executed utilizing traditional brewery equipment; however, the final product is often characterized by the presence of off-flavors imparted by the wort [7]. According to Rettberg et al. non-alcoholic beers (NABs) produced by restricted fermentations exhibited the highest levels of worty aroma and taste with a high level of sweetness, while NABs that underwent physical dealcoholization were the sourest, and had the least sweet taste, resembling alcoholic beer in these sensory variables. On the other hand, dealcoholized beers had less viscosity and had the most thin body, along with the lowest intensity of taste and aroma [8]. Notably, the method of dealcoholization had a minimal effect on the overall flavor profile [8]. Blending alcoholic beer with dealcoholized beer was found to produce non-alcoholic beers with a more pleasant and balanced flavor [8].

### 1.2. Dealcoholization Processes of Beer

Dealcoholization, the production of non-alcoholic beers from regular beer, involves many challenges and compromises which affect flavor. In this process, the goal is preserving the original taste of ordinary beer, but aroma losses are non-negligible and production costs can increase significantly [4]. Thermal distillation treatments need to be performed at low temperatures to preserve components sensitive to heat, and, thus, application of a vacuum is important in this process [4]. In the case of dealcoholized wines, membrane (reverse osmosis) treatment had a smaller impact on aroma and other sensory quality compared to vacuum distillation [9].

Dealcoholization via membrane-based separation has several advantages over thermal concentration processes, including low energy consumption, high efficiency and selectivity, and minimal degradation of initial feed components due to mild temperatures [10]. This usually involves a pre-concentration step followed by diafiltration (flushing out excess alcohol with water). The result is a low alcohol content concentrate which can be diluted back to the original feed volume, thereby reducing the alcohol content by up to two orders of magnitude (depending on the diafiltration water volume). The temperature during this separation process is low, usually below 10 °C. The downside is aroma losses: volatile esters, aldehydes pass through the membrane in significant amounts, even in the case of reverse osmosis [4,11]. In the case of osmotic distillation, Liguori et al. observed 99% losses for aromatic esters [12]. Osmotically-driven membrane processes provide an interesting alternative for the future [13], but at the moment pressure-driven processes dominate the state-of-the-art applications in the food industry [14,15,16].

As a compromise between aroma retention and alcohol passage, the use of polyamide loose RO and tight NF membranes for dealcoholization has become a staple in the industry in the past decade [17]. By using tighter membranes, more aroma compounds are retained, but alcohol rejection increases as well, while looser membranes lead to higher volatile aroma losses [10]. A high aroma/ethanol selectivity and a sufficiently high ethanol flux characterize and ideal membrane for this process. According to Catarino et al., cellulose acetate had a superior (lower) ethanol/water selectivity compared to polyamide membranes [18], but as polyamide membranes developed and dominated the RO/NF market, they are now most commonly applied for this purpose as well. Espinosa et al. found the tight NF99 HF membrane the most optimal for bitter extract/alcohol separation [19]. During the dealcoholization of white wine, Labanda found that loose RO and tight NF yield similar aroma rejection values [20], and Banvolgyi et al. successfully reduced the alcohol content of red wine via nanofiltration while maintaining similar sensory attributes as the original wine [21]. Polyphenol rejection is strongly dependent on MWCO in the case of NF and tight UF, the optimal pore size depending on the compounds needed to be retained [22].

RO requires high pressures for an optimal dealcoholization process [18], so it is valuable to test various NF membranes which might have better aroma/alcohol selectivity, while having significantly lower transmembrane pressure (TMP) demand.

### 1.3. Polyelectrolyte Multilayer (PEM) Membranes

Polyelectrolyte multilayer (PEM) membranes represent a unique type of NF membrane, which are produced by the layer-by-layer method (LbL) [23,24,25,26]. Polycations and polyanions are alternatingly layered on a charged substrate (typically an ultrafiltration membrane) (UF), building a dense amorphous polyelectrolyte multilayer (PEM), which acts as the active separation layer. The PEM has a high charge density which leads to high salt passage, a relatively greater rejection of organics, and typically high mono/divalent salt selectivity [23,24,27,28,29,30,31]. It is known that extremely high selectivity can lead to negative rejections in the case of NF membranes [32,33,34,35], and this has been observed in PEM NF membranes as well [31,36,37,38]. Besides high selectivity, PEM membranes have further advantages compared to polyamide NF membranes, with a high resistance to oxidants, such as chlorine, a higher pH tolerance (in the case of strongly acidic/basic polyelectrolytes) [39,40], high self-healing ability [41,42,43] and, if the substrate allows, solvent resistance [44,45]. The LbL method lends itself to produce NF membranes with a hollow fiber geometry by using UF membranes with such a geometry as substrates. Hollow fiber membranes have distinct advantages, including a higher crossflow tolerance, no spacer fouling, and the option of backwashing [46,47]. On the other hand, in the case of longer fibers, concentration polarization can cause significant issues [48,49], certain feed conditions can destabilize the top layer [25] (very high salinity, surfactants), and the maximum transmembrane pressure recommended by the manufacturers is only 6 bar (for Pentair HFW1000 and NX Filtration dNF membranes).

For our dealcoholization study, we chose the tightest commercially available polyelectrolyte multilayer membrane, namely dNF40 from NX Filtration, which has a nominal molecular weight cutoff (MWCO) value of 400 Da and a minimal MgSO_4_ rejection of 93%. These membranes have shown exceptional selectivity for volatile fatty acid separation [50], micropollutant rejection [51], and mono/divalent salt separation [30].

### 1.4. Ion Rejection of PEM NF Membranes in Complex Matrices

Feed composition is well known to affect ion rejections of NF and RO membranes. The influence of electrostatic effects is known to affect rejection simply by changing the feed concentration of simple salts [52,53]. Even without organic foulants, the effect of pH and salt concentrations on the rejection and flux of NF membranes is a complex phenomenon, which is further modified by the effect of foulants [54].

Pore tightening can also occur from organic fouling, as shown by Elimelech et al. [55]. Colloidal fouling can produce “cake-enhanced osmotic pressure” which decreases flux and, in relation to this, ion rejection as well [55,56]. The combination of divalent ions (Ca, Mg) with natural organic matter greatly affects the effect of organic fouling, forming a complex which forms a tight layer on the membrane surface [57]. The PEM layers on conventional RO and NF membranes are known to greatly reduce membrane fouling [58,59,60,61]. The study by Virga et al. demonstrates that fouling also has a significant effect on the divalent ion rejection of PEM NF membranes [62]. The charge inversion phenomenon examined by de Vos and Lindhoud provides a good explanation for this effect [63].

### 1.5. Low Rejection Compounds Affecting Dealcoholized Beer Taste

PEM NF membranes are well known to have a high inorganic salt passage; the salt loss can have a substantial effect on the taste of treated beverages. The presence of sodium has a discernible impact on the perceived mouthfeel of beer. While a maximum level of 50 parts per million is generally recommended, certain beer styles (particularly in darker beers) may benefit from elevated levels of sodium obtained through the addition of table salt or baking soda [64,65,66]. Chloride (by the addition of NaCl or CaCl_2_) has been utilized in brewing to enhance the perceived mouthfeel, creaminess, and overall character of beers [64,66]. Optimal calcium ion levels are important for the brewing process; magnesium can enhance the bitterness, but above 30 ppm it can add astringency [64,66].

Glycerol also plays an important role in contributing to the sensory characteristics, body, and flavor of beer [67]. Having a molecular weight of only 92 Da, it is expected to pass through NF membranes in large quantities, but a significant concentration reduction is also expected in the case of loose RO membranes, with typical MWCO values being less than 200 Da [68].

Post dealcoholization aromatization is also a viable strategy to improve flavor, which has been successfully tested for wines [69] as well as beer [70]. Ramsey et al. found that pre-processing factors, such as the raw materials used, and post-brewing processes, such as the use of additive flavor compounds or dry hopping, can have a greater influence on the overall quality of non-alcoholic beers, challenging previous findings that production methods (limited fermentation, type of dealcoholization process) were the main factor [71]. Dry-hopping was identified as a simple method to produce non-alcoholic beers with a more well-balanced flavor, even though it may be atypical for a pilsner-style beer [8]. Blending extracted aroma compounds obtained by pervaporation treatment before the dealcoholization step has also been proven to produce a non-alcoholic beer with an ethanol content lower than 0.5 vol.% that has a flavor profile very similar to the original beer [70].

### 1.6. Aims of This Study

In our study, we tested the dealcoholization of beer using the tightest commercially available PEM NF membrane, which has a nominal MWCO of 400 Da, which is considered loose for this purpose, raising questions about its applicability. On the other hand, the high organic rejection characteristic of PEM NF membranes validated the trial. We took careful measures to obtain samples for sensory evaluation, in order to provide a proof-of-concept that this membrane type is viable for this purpose. The hollow fiber geometry of the applied membrane enabled the direct dealcoholization of unfiltered beer, so we compared the membrane separation process of two batches of lager beer, filtered and unfiltered. Due to the low salt rejection of PEM membranes, we monitored the ion losses and examined the effect of re-salting (and glycerin addition) on the taste of the obtained samples.

## 2. Materials and Methods

### 2.1. Membrane Pilot Equipment and Experiments

#### 2.1.1. Closed-Circuit Membrane Test System

**Figure 1:**
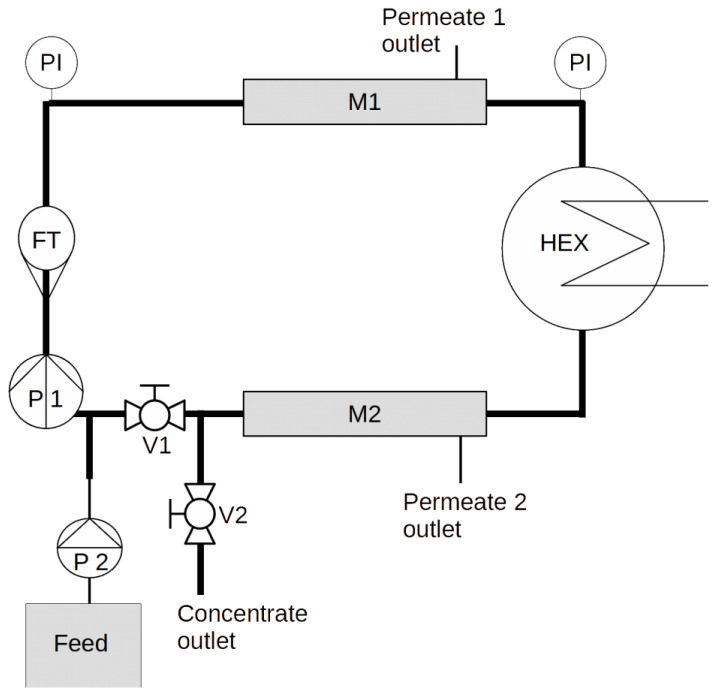
Closed-circuit small pilot experimental setup (legend in text).

A custom made (designed by the authors, manufactured by GYGV Ltd., Nagykanizsa, Hungary) closed-circuit small pilot system (see Figure 1) was used to carry out the experiments. The piping is DN20 AISI316 stainless steel, and V1 and V2 are stainless steel ball valves.

The system is capable of accepting up to two membrane modules (denoted M1 and M2). The MP025/RX300 membrane modules (300 × 25 mm hollow fiber or tubular modules) are enclosed with AISI316L stainless steel endcaps (supplied by GYGV Ltd., Hungary), with ITV STX super-rapid fitting stainless quick connectors, connected to 6/4 Teflon tubing, which serve as the permeate outlets.

Crossflow flow rate is measured with a Siemens FM100 magnetic flow transmitter (FT), which also measures the temperature of the internally circulating fluid. Crossflow velocity is maintained by adjusting the speed of a small stainless steel centrifugal pump (Shurflo COMSV024D), denoted as P1. The inner temperature is controlled by circulating cooling water from a 100 L thermostated tank by a small plastic centrifugal pump, through a stainless steel (AISI 316) Wagner OVB 20 kW tubular heat exchanger. Pressure is measured by two analog manometers and logged by hand.

During filtration mode, V2 is closed and V1 is opened; during flush mode this is reversed (the latter was not applied in the experiment described in this article).

This setup allows the direct control of the permeate flux by using a positive displacement pump as a feed pump (denoted P2). Similarly to large-scale membrane systems, the variable is the transmembrane pressure and not the permeate flow during the separation process. For this experiment, we used a Grundfos DDA dosing pump, which enables the precise control of the feed flow at pressures up to 10 bar.

#### 2.1.2. Dealcoholization Process

The M1 and M2 membrane modules used for the experiment were dNF40 membranes (MWCO ≈ 400 Da) supplied by NX Filtration BV., in MP025 membrane modules (300 × 25 mm size, 110 pcs of 0.7 mm ID hollow fibers, 0.05 m^2^ each), originating from different production batches (M1 was produced in 2021, M2 in 2022).

The inner temperature of the circulating fluid was kept at 19 ± 1.2 °C. A crossflow velocity of 0.6 m/s (90 L/h) was maintained by adjusting the circulation pump (P1) speed, at which the pressure drop over one membrane module was approximately 0.1 bar; this was taken into consideration when calculating the transmembrane pressures. The internal volume of the system is 1.65 L, but it was only filled with 1.6 L to leave gas space to act as a pulse dampener. The system was sterilized by circulating pH = 12.5 40 °C NaOH solution, and was then washed with 40 °C ultrapure water.

Beer samples (filtered and unfiltered Pécsi Prémium Lager) were supplied by Pécsi Brewery. Measures were implemented to ensure an oxygen-free environment to protect the beer aroma compounds from oxidation. After draining the system (by opening V2 and removing a manometer), it was washed and filled with 5.0 purity argon gas. It was filled with the feed pump with 1.6 L beer and closed by reinstalling the manometer. The pre-concentration phase took place in the first two hours of the experiment, when 2 L of beer was fed into the system with a constant flow rate of 1 L/h which constitutes a 10 L/m^2^h average flux through the membranes. Within a minute, the beer feed was switched to boiled and cooled (deoxygenated) ultrapure water, and feeding continued for 3 h with 1 L/h. No concentrate was released throughout the 5 h of the experiment. After 5 h, argon was fed into the top of the system by switching a manometer to the gas inlet. The concentrated dealcoholized beer was drained on the bottom through V2 into a plastic bottle filled with argon.

Permeate samples were taken; however, because there was no mode of taking concentrate (which is essentially the same as the apparent feed because of the high crossflow closed-circuit setup) samples, rejections were only calculated (according to Equation (2)) at the end point of the experiment.

#### 2.1.3. Membrane and System Cleaning

After the dealcoholization experiments, the membranes were cleaned by filling up the closed-circuit system with warm (35 °C) demineralized water, which was circulated at a high crossflow velocity (0.75 m/s) for 5 min and drained. This was repeated two times. Subsequently, the system was filled with 35 °C 0.02 M NaOH (pH = 12.3) and this was circulated at a 0.75 m/s crossflow velocity for 15 min. This was also repeated, and the system was left in this solution for 10 h. Before continuing the next experiment (dealcoholization and membrane characterization with pure water permeability and simple salt rejection measurements), the caustic was washed from the system with water, by filling up the closed-circuit system with warm (35 °C) demineralized water, which was circulated at a high crossflow velocity (0.75 m/s) for 5 min and drained. After three repetitions, during the final segment, water was fed through the feed pump with 2 L/h and the permeate let out for 60 min to thoroughly clean the permeate side as well.

#### 2.1.4. Simple Salt Rejection Measurements

After thorough cleaning with water (see previous subsection), the closed-circuit system was filled with 5 mmol/L solutions of simple inorganic salts. After determining the approximate rejection by feeding with demineralized water, the feed was set to a concentration of the estimated permeate concentration, thereby enabling an equilibrium state during the measurement. Temperature was set to 20 ± 1.5 °C. Transmembrane pressure was set to 3 bar, and crossflow velocity to 0.5 m/s. Concentrations were determined by conductivity measurements.

### 2.2. Sample Analysis

The pH was measured by a Hanna Instruments HI9812-5 portable unit, while conductivity was measured by a benchtop Consort C3210 unit with SK12T electrodes. The beer analysis (real extract, alcohol content) was performed using an Anton Paar Alcolyzer Plus system. Ion chromatography measurements were carried out on a Thermo Scientific Dionex Aquion system equipped with an AS-DV autosampler. Samples were diluted 1:25 with demineralized water in plastic vials, and non-permeate samples were filtered with 0.22 μm PTFE filter cartridges. The uncertainty of the IC measurements was ±5%.

### 2.3. Calculations

The permeance was calculated in liters per square meter per hour per bar (L/m^2^h bar) by applying the following Equation (1):

(1) $$k=\frac{\Delta V}{Am*\Delta t*p}$$

where *k* is the permeance, Δ*V* is the total volume of the permeate (L) collected at Δ*t*, the filtration time (h), *Am* is the effective membrane filtration area (m^2^), and *p* is the applied transmembrane pressure.

The observed rejection was calculated using Equation (2), as follows:

(2) $$R_{i}\left(\%\right)=\frac{{C_{f}}_{i}-{C_{p}}_{i}}{{C_{f}}_{i}}*100$$

where *C_f_* and *C_p_* represent the solute content in the feed and permeate streams, respectively.

### 2.4. Sensory Evaluation

Descriptive sensory evaluation based on the IFS Food Standard was carried out on four samples per source type (filtered or unfiltered Pécsi Prémium Lager). First, the undiluted concentrate was tested. Then, a batch of concentrate was diluted with carbonated demineralized water in 1:1 ratio. Afterwards, 22.5 mg NaCl and 325 mg KCl were added to 500 mL of the diluted carbonated sample. Glycerol was added to the salted samples (0.5 g to 250 mL) for the final sensory evaluation.

## 3. Results

### 3.1. Dealcoholization Process

**Figure 2:**
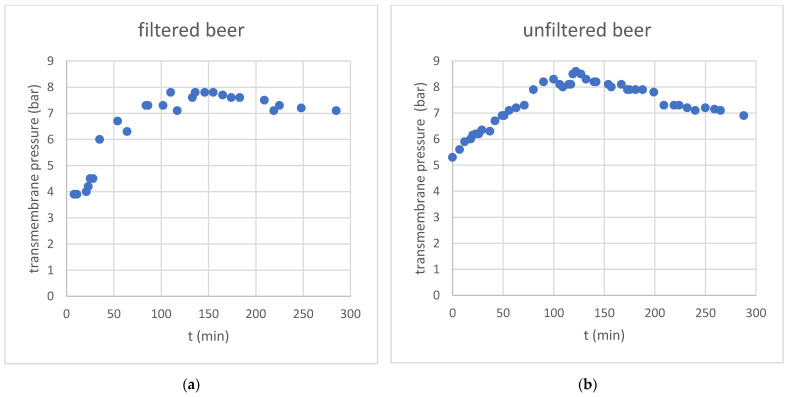
Transmembrane pressure of the beer dealcoholization process (concentration and diafiltration) at constant permeate flux rate for (**a**) filtered beer and (**b**) unfiltered beer.

Due to the constant feed flux, the transmembrane pressure increased steadily during the concentration phase in the first 120 min of the experiment (see Figure 2). After switching to water feed in the diafiltration phase, the pressure decreased steadily, although by not as much as it would have been expected by the conductivity value.

The original unfiltered lager sample was more concentrated and, therefore, resulted in a higher TMP.

In Table 1 and Table 2, the properties of the untreated beer and final non-alcoholic beers can be compared. The alcohol passage is ~100% (even a slightly negative alcohol rejection can be observed within measurement errors), which is ideal for dealcoholization purposes. The re-diluted concentrates, which are the dealcoholized end product, contain less than 0.5% alcohol. The similar pH of the permeates indicates a high organic acid passage, but the pH change in the end product is not substantial. The rejection of real extract compounds is considerable, leading to extract losses of 15% for the filtered and 18% for the unfiltered beer samples.

The high passage of Na and K ions (around 50%—see Table 3) lead to significant losses (approx. 80% decrease) in the product. By contrast, a less than 15% loss of Ca ions and an approximately 20% loss of Mg ions could be observed.

### 3.2. Sensory Evaluation

#### 3.2.1. Samples Originating from the Unfiltered Beer Concentrate

- Dealcoholized concentrate: pleasant, malty odor, with a pleasantly sweet taste and a slight lingering adhesive bitter aftertaste.
- The 1:1 diluted concentrate: the pleasant malty odor remained, although the adhesive bitterness decreased.
- After NaCl and KCl addition: pleasant malty taste, perhaps a bit fuller than the previous one, but a bit too salty.
- After NaCl, KCl, and glycerin addition: fuller and rounder flavor than the previous one, with a slight salty taste in the background.

#### 3.2.2. Samples Originating from the Filtered Beer Concentrate

- Dealcoholized concentrate: less distinctive aroma than the yeasty beer, slightly sweet and hop-like, clinging bitterness.
- The 1:1 diluted concentrate: dilution further decreased the aroma, reminiscent of cotton candy. The diluted beer had a distinctly watery taste, with only bitterness being perceivable, but no fullness or sweetness.
- After NaCl and KCl addition: salting did not affect the aroma but complemented the taste; in addition to the bitterness, some fullness was also noticeable.
- After NaCl, KCl, and glycerin addition: out of the last four samples, this one was the best from the sensory point of view, and the addition of glycerin increased the fullness of the flavor.

## 4. Discussion

### 4.1. Dealcoholization Process

The average permeate flux of 10 L/m^2^h can be considered a bit low, but we were already close to the operational limits of the dNF40 membrane. The applied transmembrane pressure exceeded the 6 bar maximum advised by NX Filtration, and the maximum of 8.6 bar was close to the maximum allowed pressure of 10 bar. Moreover 6 bar is the allowed maximum for the larger full-scale modules produced at the writing of this article.

The temperature of 19 °C is relatively high for such a process, considering the industrial standard is <10 °C for conventional LPRO. On the other hand, rejection properties of PEM NF membranes do not deteriorate as much as can be observed with polyamide membranes; therefore, the optimal temperature for this type of membrane might be higher than for polyamide membranes. The high losses of Na, K, and Cl ions need to be considered because this can detrimentally effect product quality. On the other hand, divalent ion rejections are sufficiently high; furthermore, if the dilution is not 1:1.125, but 1:1 (the one we used for sensory examination), then the final product practically has no change in divalent cation concentration.

Mg rejection was observed to be two times higher (99% vs. 98%) than Ca, which is typical for NF membranes, but the opposite can be observed for negatively charged PEM membranes. In fact, for the latter, magnesium rejection can be significantly lower compared to monovalent sodium ions.

### 4.2. Membrane Cleaning and Membrane Characteristics after the Process

**Figure 3:**
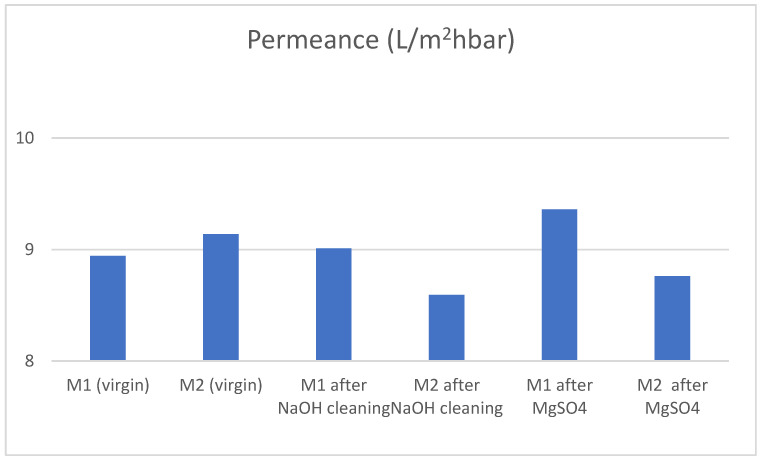
Permeance of virgin and cleaned membranes.

The membrane was successfully cleaned by caustic cleaning at a pH of 12.3. Permeance was efficiently recovered after the cleaning process, with non-significant changes (see Figure 3). Salt rejections increased notably (see Table 4), especially in the case of MgSO_4_. This suggests that the strongly negative charge of the membrane surface decreased, thereby increasing the rejection of the divalent Mg ion compared to Na.

The increase in rejection while maintaining permeance is a notable improvement which substantiates further investigation of this phenomenon in the future.

### 4.3. Product Quality

Unfiltered beer can also be directly dealcoholized with this process. Yeast have been observed to adsorb a variety of aromatic compounds present in beer during fermentation, which impacts the overall flavor and aroma profile of the final product. The adsorbed compounds are retained with the yeast cells during the membrane separation process, boosting the aroma content of the concentrate. This was supported by our sensory evaluation result, in which the unfiltered beer received more positive descriptions.

Summarizing the results of the sensory evaluation, we suggest the addition of mineral substances and glycerin after alcohol removal with PEM NF membranes. The sensory properties are enhanced by salt replenishment, masking the empty or overly bitter taste, although the amount of sodium added was a bit over the optimal level. By reducing the NaCl, the ratio of sodium and potassium will become balanced. Adding glycerin greatly improved the flavor, resulting in a full and rounded out taste for the beer.

We suggest the study of mineral and glycerol replacement for other membrane-based dealcoholization methods also performed by tight NF or loose RO polyamide membranes. Potassium generally has a lower rejection for polyamide membranes, and the passage of glycerol could also significantly affect the taste. The addition of hops aroma or dry-hopping after the dealcoholization process could also improve the final product by replacing the lost volatile aroma compounds.

## 5. Conclusions

We successfully dealcoholized two samples of Pécsi Prémium lager beer (filtered and unfiltered) with the commercially available hollow fiber polyelectrolyte membrane, dNF40, at a small pilot scale. This study provides evidence of the successful dealcoholization of unfiltered beer for the first time using a pressure-driven membrane process, and the first-ever application of PEM NF membranes in food processing. Even though the nominal pore size of the applied membrane can be considered high for this purpose, sensory evaluation showed satisfactory results after replenishing lost salts and glycerin. The transmembrane pressure in our experiment was close to the pressure tolerance limits of this particular membrane, but the advantages offered by PEM NF membranes, such as directly treating hazy unfiltered beer and high permeability, justify further research in this area.

Lots of questions remain open for future investigations, such as the following

- Testing the effect of the temperature of the membrane separation process on taste;
- Instead of direct preconcentration, starting with dilution of the feed, which could help overcoming pressure limits. This leads to longer processing times, although PEM NF membranes are known to swell in higher salinity feeds, which lowers the rejection of organics;
- Testing tighter and/or more pressure tolerant PEM-NF membranes;
- Optimizing the salt and glycerol amount added. The addition of hop aroma concentrates to the final product can further enhance the aroma of these beers;

There are many variables yet to optimize, which holds the possibility of creating a novel technology for beer dealcoholization.

## Figures and Tables

**Table 1:** Conductivity, pH, alcohol, and real extract content of original and dealcoholized beer and permeate samples.

Sample	Conductivity (μS/cm)	pH	Alcohol (% *v*/*v*)	Real Extract (% *w*/*w*)
Filtered beer	1880	4.4	4.7	3.64
Unfiltered beer	1940	4.5	5.0	3.90
Dealcoholized beer filtered	698	4.3	0.4	3.10
Dealcoholized beer unfiltered	797	4.4	0.4	3.21
Average permeate from concentration, filtered feed	1196	4.4	4.9	0.41
Average permeate from concentration, unfiltered feed	1225	4.5	5.1	0.42
Average permeate at the end of the process, filtered feed	545	4.3	0.9	0.16
Average permeate at the end of the process, unfiltered feed	562	4.5	0.9	0.14

**Table 2:** Inorganic cation and anion concentrations in original and dealcoholized beer and permeate samples.

Sample	Na^+^ (ppm)	K^+^ (ppm)	Ca^2+^ (ppm)	Mg^2+^ (ppm)	Cl^−^ (ppm)	PO_4_^3−^ (ppm)	SO_4_^2−^ (ppm)
Filtered beer	17	485	103	24	216	660	62
Unfiltered beer	16	583	110	22	241	651	123
Deal. filtered	3	82	93	18	14	425	56
Deal. unfiltered	4	127	94	19	19	362	103
End point permeate of filtered M1	5	141	1	1	36	146	3
End point permeate of filtered M2	5	148	2	1	53	101	1
End point permeate of unfiltered M1	4	150	1	0	33	134	2
End point permeate of unfiltered M2	4	148	1	1	43	87	0

**Table 3:** Salt rejections at the end of the dealcoholization process.

Feed, Membrane	Na^+^	K^+^	Ca^2+^	Mg^2+^	Cl^−^	PO_4_^3−^	SO_4_^2−^
Filtered beer, M1	19%	24%	99%	98%	−27%	82%	97%
Filtered beer, M2	16%	20%	99%	97%	−48%	31%	69%
Unfiltered beer, M1	54%	47%	>99%	99%	22%	84%	99%
Unfiltered beer, M2	52%	48%	>99%	99%	−33%	35%	83%

**Table 4:** Pure salt rejections before and after cleaning.

Title 1	MgSO_4_ Rejection	Na_2_SO_4_ Rejection
M1, virgin	96%	98%
M2, virgin	95%	99%
M1, after cleaning	98.8%	99.6%
M2, after cleaning	98.9%	99.2%

## Data Availability

The data presented in this study are available on request from the corresponding author.
